# Neoadjuvant chemotherapy and trastuzumab versus neoadjuvant chemotherapy followed by post-operative trastuzumab for patients with HER2-positive breast cancer

**DOI:** 10.18632/oncotarget.4801

**Published:** 2015-08-21

**Authors:** Carlo Palmieri, Iain RJ Macpherson, Kelvin Yan, Felipe Ades, Pippa Riddle, Riz Ahmed, Waheeda Owadally, Barbara Stanley, Deep Shah, Ondrej Gojis, Adam Januszewski, Conrad Lewanski, Rebecca Asher, Daniel Lythgoe, Evandro de Azambuja, Mark Beresford, Sacha J. Howell

**Affiliations:** ^1^ Academic Department of Medical Oncology, Clatterbridge Cancer Centre NHS Foundation Trust, Wirral, UK; ^2^ Liverpool and Merseyside Academic Breast Unit, The Linda McCartney Centre, Royal Liverpool University Hospital, Liverpool, UK; ^3^ The University of Liverpool, Department of Molecular and Clinical Cancer Medicine, Institute of Translational Medicine, Liverpool, UK; ^4^ Department of Medical Oncology, Beatson West of Scotland Cancer Centre, Glasgow, UK; ^5^ Department of Medical Oncology, Imperial College Healthcare NHS Trust, London, UK; ^6^ Department of Medical Oncology, Institut Jules Bordet, Université Libre de Bruxelles, Brussels, Belgium; ^7^ Department of Clinical Oncology, West Middlesex University Hospital, London, UK; ^8^ Department of Clinical Oncology, The Royal United Hospital, Combe Park, Bath, UK; ^9^ Ealing Hospital NHS Trust, Middlesex, UK; ^10^ Cancer Research UK Liverpool Cancer Trials Unit, Liverpool, UK; ^11^ University of Manchester, Institute of Cancer Sciences, Department of Medical Oncology, The Christie NHS Foundation Trust, Manchester, UK

**Keywords:** breast, neoadjuvant, trastuzumab, concomitant, sequential

## Abstract

Neoadjuvant chemotherapy plus trastuzumab (NCT) increases the rate of pathological complete response (pCR) and event-free survival (EFS) compared to neoadjuvant chemotherapy (NC) alone in women with HER2 positive breast cancer (BC). pCR in this setting is associated with improved EFS. Whether NCT preferentially improves EFS in comparison to NC followed by adjuvant trastuzumab initiated postoperatively (NCAT) has not been addressed. Using clinical data from women with HER2 positive BC treated at 7 European institutions between 2007 and 2010 we sought to investigate the impact on breast cancer outcomes of concomitant (NCT) versus sequential (NCAT) treatment in HER2 positive early BC. The unadjusted hazard ratio (HR) for event free survival with NCT compared with NCAT was 0.63 (95% CI 0.37–1.08; *p* = 0.091). Multivariable analysis revealed that treatment group, tumour size and ER status were significantly associated with EFS from diagnosis. In the whole group NCT was associated with a reduced risk of an event relative to NCAT, an effect that was confined to ER negative (HR: 0.25; 95% CI, 0.10–0.62; *p* = 0.003) as opposed to ER positive tumours (HR: 1.07; 95% CI, 0.46–2.52; *p* = 0.869). HER2 positive/ER negative BC treated with NC gain greatest survival benefit when trastuzumab is administered in both the neoadjuvant and adjuvant period rather than in the adjuvant period alone. These data support the early introduction of targeted combination therapy in HER2 positive/ER negative BC.

## INTRODUCTION

Adjuvant trastuzumab (AT) improves overall survival (OS) in HER2-positive early breast cancer when administered concomitantly with, or sequentially after chemotherapy [1, 2]. In the neoadjuvant setting the addition of trastuzumab to chemotherapy (NCT) has been shown to increase pathological complete response (pCR) compared with neoadjuvant chemotherapy (NC) alone [3, 4, 5], and translated to a non-significant improvement in breast preservation favouring NCT over NC [6]. NCT also resulted in significant improvements in disease-free survival (DFS) [3] and event free survival (EFS) [4, 5] although very few patients in the NC control arms received AT in either study [0% and 17%] reflecting the standard of care at the time [3, 4]. These studies do not, therefore, address whether there is an advantage to commencing trastuzumab concomitantly with neoadjuvant chemotherapy or whether trastuzumab may be administered after completion of neoadjuvant chemotherapy and surgery with equal efficacy. We have previously reported that DFS may be inferior to the published data in a group of patients treated in the neoadjuvant setting where the trastuzumab was commenced postoperatively [7].

In the adjuvant setting, studies that employed concurrent versus sequential trastuzumab and chemotherapy were associated with lower hazard ratios for DFS, and the only negative adjuvant study used a sequential strategy [8]. However, only one study, North Central Cancer Treatment Group (NCCTG) N9831, directlty addressed the sequencing question; this randomised women to AC-paclitaxel chemotherapy with or without trastuzumab either in sequence or combination with the paclitaxel portion of the regimen. Improved DFS was observed in the concurrent vs sequential arm, although this did not reach prespecified levels of statistical significance [9]. Based on the suggested improved efficacy without any significant increase in toxicity, concurrent trastuzumab and taxane chemotherapy was suggested to be a new standard of care.

As the effect of sequencing trastuzumab within the context of neoadjuvant therapy has not been addressed in prospective trials we sought to compare the EFS and OS of patients treated with NCT versus those receiving sequential NCAT in a multicentre retrospective cohort.

## RESULTS

In total, 236 eligible patients were identified, of whom 98 had received neoadjuvant chemotherapy with concomitant trastuzumab (NCT) the latter being completed post-operatively and 138 who received neoadjuvant chemotherapy alone with the trastuzumab commenced post-operatively (NCAT). For all patients the planned duration of trastuzumab was one year. Baseline clinico-pathological features are summarised in Table 1. The groups were balanced in terms of age, histological type, tumour size and estrogen receptor (ER) status, but differed with regards to chemotherapy regimen and tumour grade.

**Table 1:** Baseline demographics

Baseline Demographics				
Variable	All patients (*n = 236*)	NCT (*n = 98*)	NCAT (*n = 138*)	*p*-value
Age	50.4 (10.9)	51.4 (11.0)	49.7 (10.8)	0.2506
Histological type				
IDC	223 (94.5)	93 (94.9)	130 (94.2)	
Other	13 (5.5)	5 (5.1)	8 (5.8)	0.529
Tumour size				
<20 mm	16 (6.8)	3 (3.1)	13 (9.4)	
20–50 mm	146 (61.9)	62 (63.3)	84 (60.9)	
>50 mm	57 (24.2)	25 (25.5)	32 (23.2)	
Inflammatory/Others	17 (7.2)	8 (8.2)	9 (6.5)	0.278
Tumour grade				
1–2	75 (31.8)	21 (21.4)	54 (39.1)	
3	135 (59.2)	58 (57.2)	77 (55.8)	
Not Available	26 (11.0)	19 (19.4)	7 (5.1)	**<0.001**
Estrogen receptor status				
Positive	124 (52.5)	50 (51.0)	74 (53.6)	
Negative	111 (47.0)	48 (49.0)	63 (45.7)	
Not Available	1 (0.4)	0	1 (0.7)	0.632
Chemotherapy regimen				
Anthracycline	98 (41.5)	26 (26.5)	72 (52.2)	
AnthracyclineTaxane	118 (50.0)	58 (59.2)	60 (43.5)	
Taxane	5 (2.1)	3 (3.1)	2 (1.5)	
Other	15 (6.4)	11 (11.2)	4 (2.9)	**<0.001**

The median time to surgery from diagnosis was 5.72 months (IQR: 5.22–6.44). Time to surgery was significantly longer (*p* = 0.012) for the NCT group (5.95 months; IQR 5.35–6.77) in comparison to the NCAT group (5.52 months; IQR 5.03–6.21), (Table 2). As expected, the time from diagnosis to loading dose of trastuzumab was significantly shorter for the NCT group (3.5 months, IQR 2.96–4.04) as compared to NCAT group (8.4 months; IQR 6.64–9.63), *p* < 0.001 (Table 2).

**Table 2:** Summary of follow-up time, surgery and trastuzumab treatment

	Overall	NCT	NCAT	*p*
Follow-up	53.7 (41.7, 68.8)	44.8 (37.0, 53.9)	61.5 (50.3, 78.5)	
Time to first traz	6.04 (3.52, 8.71)	3.45 (2.96, 4.04)	8.38 (6.64, 9.63)	**<0.001**
Time to surgery	5.72 (5.22, 6.44)	5.95 (5.35, 6.77)	5.52 (5.03, 6.21)	**0.012**
Trastuzumab Status:				
Completed	185 (78.4)	78 (80.0)	107 (77.5)	
Ongoing	13 (5.5)	11 (11.2)	2 (1.5)	
Stopped	38 (16.1)	9 (9.2)	29 (21.0)	**<0.001**
Reason for stopping:				
Cardiotoxicity	15 (39.5)	3 (33.3)	12 (41.4)	
Patient's decision	2 (5.3)	1 (11.1)	1 (3.5)	
Relapse	15 (39.5)	4 (44.4)	11 (37.9)	
Other	3 (7.9)	1 (11.1)	2 (6.9)	
Unknown	3 (7.9)	0	3 (10.3)	0.712
Number of Trastuzumab cycles:	18 (1–36)	18 (2–36)	18 (1–20)	0.8623

At the time of analysis, treatment had been completed in 185 (78.4%) patients, was still ongoing in 13 (5.5%) and had been stopped before completion for 38 (28%). Of those cases that stopped treatment prematurely; 15 were due to cardiotoxicity (NCT 3 vs NCAT 12), 15 to relapse (NCT 4 vs NCAT 11), 2 to the patient's decision (NCAT 1 vs NCT 1) and 3 for other reasons (NCT 1 vs NCAT 2). The status of trastuzumab treatment differed significantly between groups (*p* < 0.001) with a higher proportion still ongoing treatment in the NCT group and a higher proportion stopping before completion in the NCAT group (Table 2). The number of trastuzumab cycles received did not differ between groups.

At the median follow up of 53.7 months, a total of 49 events and 39 deaths had occurred. Fewer events were seen in the NCT group than the NCAT group [NCT 15 (31%) vs NCAT 34 (69%)] and of these events 43 (88%) were relapses; 14 (33%) in the NCT group and 29 (67%) in NCAT group. Of the 39 deaths, 33 (85%) were due to breast cancer and fewer had occurred in the NCT group than the NCAT group; 9 (23%) and 30 (77%), respectively.

**Figure 1:**
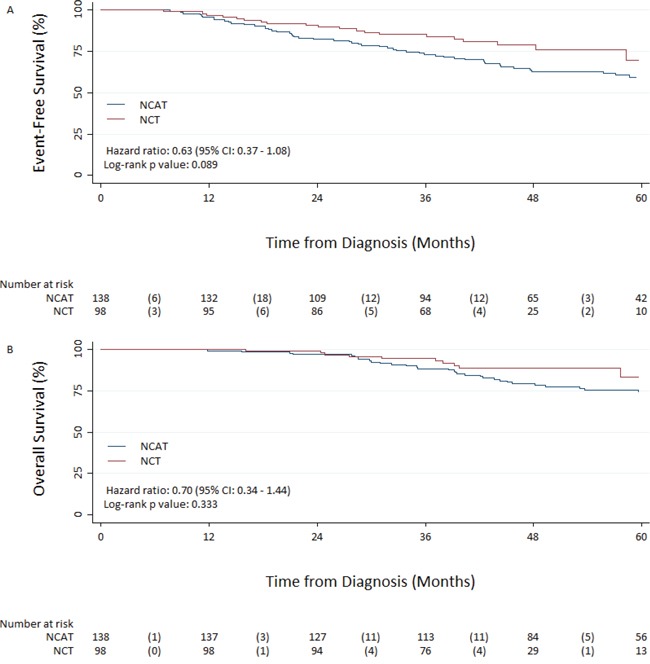
Event-free Survival A. and overall survival B. from diagnosis NCAT: Neoadjuvant chemotherapy followed by adjuvant trastuzumab; NCT: Neoadjuvant chemotherapy delivered with concomitant trastuzumab.

5-year EFS from diagnosis was 69.6% (95% CI: 51.5–82.0) in the NCT group and 59.3% (95% CI: 49.8–67.6) in the NCAT group. The unadjusted hazard ratio for risk of an event in the NCT group compared with the NCAT group was 0.63 (95% CI 0.37–1.08; *p* = 0.091) (Figure 1A). 5-year OS from diagnosis was 83.3% (95% CI: 66.2–92.2) in the NCT group and 74% (95% CI: 65.0–81.1) in the NCAT group. The unadjusted hazard ratio of death was 0.70 (95% CI 0.34–1.44; *p* = 0.333) (Figure 1B). The HR for breast cancer-specific survival favoured NCT with cancer-specific survival at 5 years being greater in the NCT than in the NCAT group (Table 3).

**Table 3:** Summary of efficacy endpoints - 5 years by treatment from diagnosis

	All patients (*n* = 236)	NCT (*n* = 98)	NCAT (*n* = 138)	Hazard Ratio	*p*
5-Year Overall Survival	77.4% (70.2, 83.1)	83.3% (66.2, 92.2)	74.0% (65.0, 81.1)	0.70 (0.34, 1.44)	0.336
5-Year Event-free Survival	63.7% (55.8, 70.5)	69.6% (51.5, 82.0)	59.3% (49.8, 67.6)	0.63 (0.37, 1.08)	0.091
5-Year Breast Cancer Specific Survival	80.3% (73.7, 86.1)	84.8% (70.8, 94.4)	77.9% (69.9, 85.0)	0.72 (0.33, 1.56)	0.408
				**Odds Ratio**	***p***
Total pCR Rate (*n* = 230)	19.6% (45)	33.3% (31)	10.2% (14)	4.39 (2.18, 8.86)	**<0.001**
Breast pCR Rate	23.3% (55)	38.8% (38)	12.3% (17)	4.51 (2.35, 8.64)	**<0.001**

With regard to ER status, 5-year EFS from diagnosis was 70.7% (95% CI: 70.7–79.2) in women with ER positive tumours and 56.5% (95% CI: 44.7–66.6) in those with ER negative tumours. The unadjusted hazard ratio for risk of an event in women with ER negative tumours as compared to those with ER positive tumours was 2.02 (95% CI 1.26–3.26; *p* = 0.004). 5-year OS from diagnosis was 85.5% (95% CI: 75.3–91.7) in those with ER positive tumours and 69.1% (95% CI: 57.7–78.0) in those with ER negative tumours. The unadjusted hazard ratio for risk of an event in those with ER negative tumours as compared to ER positive tumours was 2.97 (95% CI 1.59–5.56; *p* = 0.001). The HR for breast cancer-specific survival significantly favoured those women who had ER positive tumours (Table 4).

**Table 4:** Summary of efficacy endpoints - 5 years by estrogen receptor status from diagnosis; ER: Estrogen Receptor

	All patients (*n* = 236)	ER + (*n* = 124)	ER − (*n* = 111)	Hazard Ratio	*p*
5-Year Overall Survival	77.4% (70.2, 83.1)	85.5% (75.3, 91.7)	69.1% (57.7, 78.0)	2.97 (1.59, 5.56)	**0.001**
5-Year Event-free Survival	63.7% (55.8, 70.5)	70.7% (59.7, 79.2)	56.5% (44.7, 66.6)	2.02 (1.26, 3.26)	**0.004**
5-Year Breast Cancer Specific Survival	80.3% (73.7, 86.1)	87.5% (78.8, 93.8)	72.0% (61.9, 81.4)	3.08 (1.58, 6.00)	**0.001**
				**Odds Ratio**	***p***
Total pCR Rate (*n* = 230)	19.7% (45)	16.4% (20)	23.4% (25)	1.55 (0.81, 3.00)	0.187
Breast pCR Rate	23.3% (55)	18.6% (23)	28.8% (32)	1.78 (0.97, 3.28)	0.065

Total pCR was obtained in 45 (19.6%) cases (33% NCT and 10% NCAT) and was significantly associated with treatment, with those receiving NCT having increased odds of pCR relative to NCAT (OR: 4.39; 95% CI 2.18–8.86; *p* = < 0.001).

**Figure 2:**
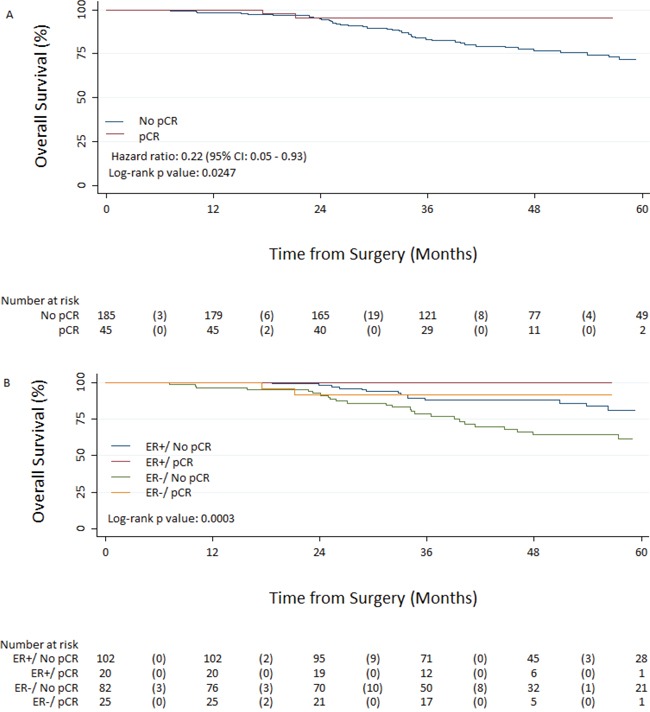
Overall survival from surgery in patients with pathologic complete response (pCR) and without pCR A. and based on Estrogen receptor status/pCR interaction B

**Figure 3:**
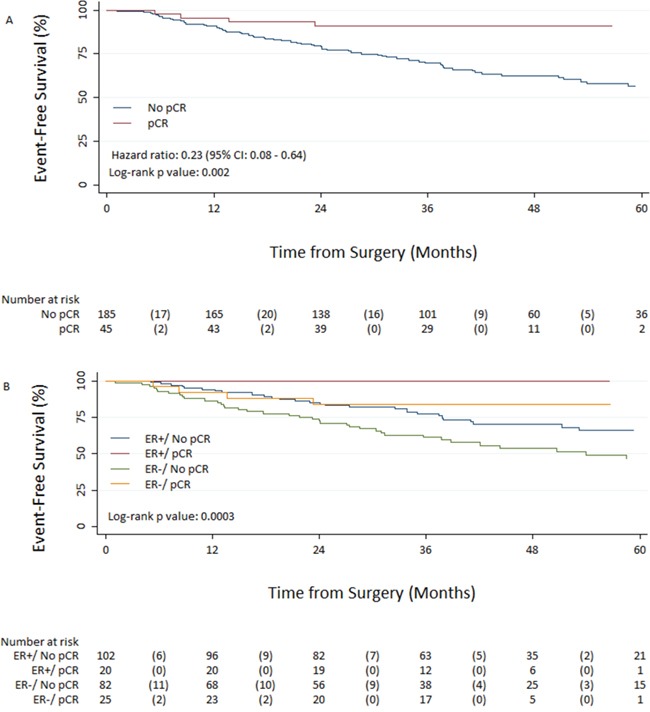
Event-free survival from surgery in patients with pathologic complete response (pCR) and without pCR A. and based on Estrogen receptor status/pCR interaction B

In the whole population, achieving total pCR was associated with significantly improved EFS from surgery, with an unadjusted hazard ratio of 0.23 (95% CI 0.08–0.64; *p* = 0.002) (Figure 2A). Total pCR was higher in women with ER negative tumours (23.4%) vs ER positive (16.4%) tumours, although this did not reach statistical significance (Table 4). However, EFS from surgery by ER status and pCR revealed a significant difference between survival curves (*p* < 0.001) (Figure 2B). Achieving total pCR was also associated with a significantly improved OS from surgery, with an unadjusted hazard ratio of 0.22 (95% CI 0.05–0.93; *p* = 0.0247) (Figure 3A). OS from surgery by ER status and pCR revealed a significant difference between survival curves (*p* < 0.0003) (Figure 3B). At surgery there was a significant difference in lymph node status between the two groups with a higher percentage of patients being node negative in the NCT group compared to the NCAT group; 68% and 39%, respectively (Table 5).

**Table 5:** Surgical variables

Surgery				
Variable	All patients (*n = 236*)	NCT (*n = 98*)	NCAT (*n = 138*)	*p*-value
**Type of Surgery:**				
Mastectomy	172 (72.9)	69 (70.4)	103 (74.6)	
Breast Conserving	60 (25.4)	28 (28.6)	32 (23.3)	
ANC only	4 (1.7)	1 (1.0)	3 (2.2)	0.537
**Residual Disease in Breast:**				
<20 mm	170 (72.0)	74 (75.5)	96 (69.6)	
20–50 mm	42 (17.8)	16 (16.3)	26 (18.8)	
>50 mm	15 (6.4)	4 (4.1)	11 (8.0)	
Not Available	9 (3.8)	4 (4.1)	5 (3.6)	0.616
**Lymph Node Status:**				
0	121 (51.3)	67 (68.4)	54 (39.1)	
1–3	59 (25.0)	16 (16.3)	43 (31.2)	
4–9	35 (14.8)	7 (7.1)	28 (20.3)	
≥10	15 (6.4)	3 (3.1)	12 (8.7)	
Missing	6 (2.5)	5 (5.1)	1 (0.7)	**<0.001**

**Figure 4:**
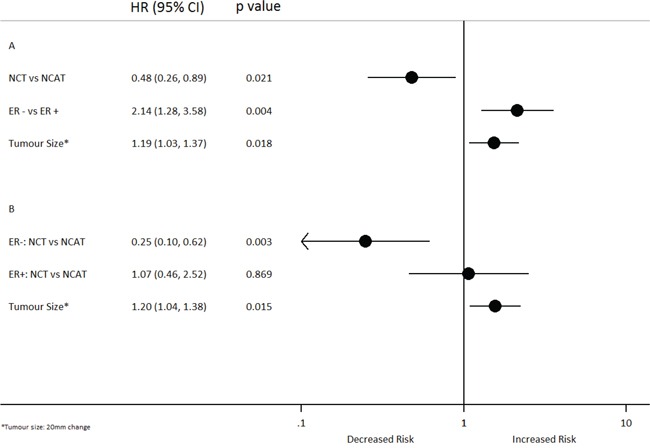
Multivariable cox regression-Event-Free Survival from diagnosis A. with interaction B. CI: Confidence Interval; HR: hazard ratio; ER:Estrogen Receptor; NCAT: Neoadjuvant chemotherapy followed by adjuvant trastuzumab; NCT: Neoadjuvant chemotherapy delivered with concomitant trastuzumab

Multivariable analysis revealed that treatment group, tumour size and ER status were associated with EFS from diagnosis. NCT was associated with a reduced risk of an event relative to NCAT (HR: 0.48; 95% CI 0.26–0.89; *p* = 0.021), whereas larger tumours and a negative ER status were associated with a significantly increased risk of an event (Figure 4A). The significant interaction between treatment group and ER status indicated that the effect of NCT vs NCAT differed by ER status; for patients with ER negative tumours, NCT was significantly associated with a reduced risk of an event relative to NCAT (HR: 0.25; 95% CI, 0.1–0.62; *p* = 0.003), which was not observed for those with ER positive tumours (HR: 1.07; 95% CI, 0.46–2.52; *p* = 0.869) (Figure 4B).

**Figure 5:**
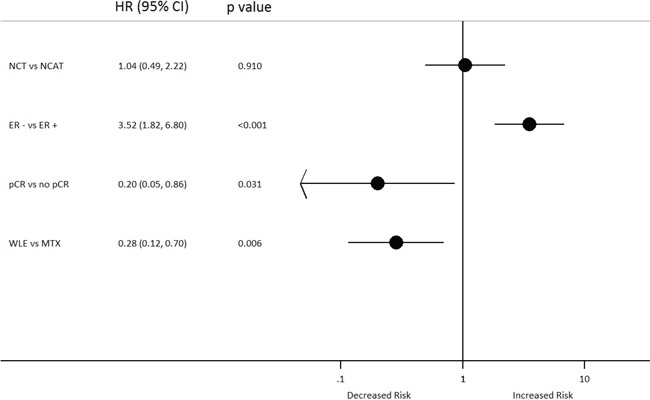
Multivariable cox regression-Overall Survival from Surgery CI: Confidence Interval; ER:Estrogen Receptor; HR: hazard ratio; NCAT: Neoadjuvant chemotherapy followed by adjuvant trastuzumab; MTX: Mastectomy; NCT: Neoadjuvant chemotherapy delivered with concomitant trastuzumab; pCR: pathologic complete response; WLE: Wide local excision.

After adjusting for pCR, ER status and surgery type, treatment group had no effect on OS from surgery. However, it was seen that a negative ER status was associated with an increased risk of death relative to a positive ER status, whereas achieving a total pCR and undergoing a wide local excision (WLE) were associated with a reduced risk of death (Figure 5).

## DISCUSSION

The concomitant use of trastuzumab with chemotherapy in the neoadjuvant setting is associated with an increased rate of pCR, however the effects on EFS and OS have been unclear when compared with delayed post-operative trastuzumab. No randomised study to date has compared the efficacy of pre- versus post-operative trastuzumab, and therefore to address this issue we present the first report of data comparing patients with HER2 positive breast cancers treated in the neoadjuvant setting where HER2 directed therapy was either commenced concomitantly with chemotherapy or in a sequential fashion following neoadjuvant chemotherapy and surgery. The sequential versus concomitant use of trastuzumab described within this study reflects the initial use of AT as per the HERA trial design [1], and the later shift to a concomitant use following data regarding the efficacy and safety of concurrent neoadjuvant chemotherapy and trastuzumab [5, 6].

The pCR rate within this study for the NCT cohort was three-fold higher than the NCAT cohort, 33% versus 10% respectively and is similar to rates observed in prospective studies [10–16]. The pCR rate was lower in the NCAT group as compared to previous prospective studies [3, 4, 11], and may reflect the more heterogeneous nature of the chemotherapy administered and the fact that not all patients received a sequential anthracycline and taxane regimen. Women with ER negative tumours experienced a higher rate of pCR than those with ER positive tumours. This is consistent with non-randomised studies of HER2 directed therapy [17–19] as well as randomised studies which have investigated chemotherapy alone versus chemotherapy plus trastuzumab [3], trastuzumab versus lapatinib [20], and trastuzumab versus lapatinib or doublet HER2 targeted therapy [10, 12, 13, 21]. In our study, pCR was associated with a significant benefit with regard to EFS and OS from surgery. These data being consistent with those from retrospective analyses, nonrandomised phase II studies, randomised studies and meta-analyses [10, 14, 15, 18, 22]. pCR following chemotherapy plus trastuzumab, lapatinib or the doublet has been shown to be a strong predictor for OS [11, 14, 15], while NOAH reported EFS to be strongly associated with pCR in patients receiving trastuzumab [5]. A recent meta-analysis of randomised neoadjuvant trials found that the effects of pCR on EFS was more marked in the HER2 positive cases with the strength of the association increased in the hormone receptor negative subgroup [22]. However, 55% of cases with HER2-positive tumours included in the meta-analysis did not receive AT as they were treated before it became standard of care. In those treated with trastuzumab, the women with hormone receptor- negative tumours achieving a pCR had the most favourable EFS and OS for all subgroups [22]. Previous studies of HER2 directed therapy within the neoadjuvant setting have not investigated the effect of sequencing HER2 therapy relative to surgery in the neoadjuvant setting, the current study being the first to investigate this question. The present findings demonstrate that the commencement of trastuzumab in the neoadjuvant setting was associated with a trend towards increased EFS and OS with hazard ratios 0.63 and 0.70 respectively. Univariable and multivariable analysis of the whole cohort showed no difference in clinical outcome with regard to the concomitant adjuvant use of trastuzumab as compared to sequential neoadjuvant use. However, significant differences in EFS were seen based on ER status, with a clear benefit from concomitant as opposed to sequential use of trastuzumab in women with ER negative disease but no benefit seen in women with ER positive tumours. It is an accepted notion that for HER2-posiitve breast cancer ER-positive disease and ER-negative disease are two distinct entities, as reflected in the differences in pCR rates in neoadjuvant setting [3, 17–21] and differences seen in patterns and incidence of relapse within adjuvant studies [23]. Possible reasons therefore for the differential benefit observed in women with ER-negative disease with regard to the concomitant use of trastuzumab include the synergistic intetraction of chemotherapy and trastuzumab including docetaxel and trastuzumab [24]. As well as the earlier introduction of targeted therapy in this high-risk group of patients. It is notable that a concomitant trastuzumab regimen resulted in exposure to trastuzumab almost 5 months earlier, on average, than sequential post-operative approach. Such delays may have been clinically significant in high risk tumour. While in women with HER2-positive, ER-positive disease the post-operative combination of endocrine therapy and trastuzumab, may have mitigated against any possible issues associated with the delayed start of trastuzumab.

The NCCTG N9831 trial is the only adjuvant study which has investigated the effect of concurrent versus sequential administration of trastuzumab with chemotherapy [9]. The estimated 5-year DFS favoured the sequential arm although this did not reach pre-specified levels of statistical significance. Interestingly, the hazard ratio with regard to EFS (HR: 0.63: 95% CI 0.37–1.08) observed in our study is similar to N9831 (HR: 0.77: 95% CI, 0.53–1.11), and favours commencing trastuzumab concomitantly with chemotherapy. As no multivariable analysis was presented within N9831, the effect of ER status on the relative benefit of concomitant versus sequential AT cannot be discerned [9].

Weaknesses of this study include the nonrandomised retrospective cohort design although randomised data addressing this question are unlikely to emerge. Overall the cohorts appeared well balanced although significant differences in the chemotherapy regimens were also noted with increased taxane use in the NCT group that may also have contributed to improved outcomes in this cohort [25]. On the other hand the true effect of NCT versus NCAT may have been underestimated as case ascertainment required patients to have received at least one cycle of trastuzumab and any patient relapsing prior to receiving the initial cycle of trastuzumab would have been excluded from the study. The time from diagnosis to first trastuzumab was significantly longer in the NCAT than the NCT group at 8.4 versus 3.4 months, potentially overestimating the efficacy of NCAT via a guarantee-time bias effect [26]. Indeed, a number of cases of relapse prior to commencing AT were noted in some of the centres involved (data not presented).

In conclusion, concomitant as compared to sequential trastuzumab is associated with improved outcomes in the neoadjuvant setting for women with ER negative/HER2 positive tumours. These data are hypothesis generating but support the concept of early initiation of targeted combination therapy in women with ER negative/HER2 positive breast cancers.

## PATIENTS AND METHODS

### Identification of HER 2-positive neoadjuvant patients

HER-2 positive patients treated with neoadjuvant chemotherapy between January 2006 and December 2011 at Imperial College Healthcare NHS Trust, West Middlesex Hospital, Ealing Hospital, Beatson West of Scotland Cancer Centre, breast units in the Greater Manchester and Cheshire Cancer Network, Bristol Oncology Centre and Institut Jules Bordet, Brussels were identified. Eligible patients had to have received at least one cycle of trastuzumab in either the neoadjuvant or adjuvant setting. Patients presenting or found to have metastatic disease were excluded.

### HER2 Positivity

HER2 positivity was determined by Immunohistochemistry (IHC) and/or Silver in-situ hybridisation (SISH) or Fluorescence in-situ hybridisation (FISH) analyses according to local protocols.

### Data collection

Data collected included patient age, date and type of surgery, histologic grade and tumour size, lymph node involvement, estrogen and progesterone receptor status, details of neoadjuvant chemotherapy and the temporal relationship to initiation of trastuzumab with regard to chemotherapy and surgery. Tumour size was based on pre-treatment measurements; radiological (mammography/ultrasound) where possible with clinical measurements used where radiological measurements were not available. Details of disease-specific relapse and death, as well as non-breast cancer deaths, were also recorded. Patients entered into the “Adjuvant Lapatinib And/Or Trastuzumab Treatment Optimisation” (ALTTO) trial were excluded from the efficacy analysis.

Event-free survival (EFS), an event was defined as new primary breast cancer, local recurrence, distant relapse or death from any cause prior to recurrence/relapse and was calculated from the date of initial diagnosis and from date of surgery. Patients without recurrence at the date of last follow-up or treatment were censored. Overall survival (OS) was defined as death from any cause and breast cancer specific survival (BCSS) was defined as death related to breast cancer; both OS and BCSS were calculated from the date of diagnosis and from date of surgery. Patients without an event were censored at the date of last follow up, telephone contact or treatment visit. Data were locked for analysis on the 14th February, 2014.

Pathological complete response was defined as the complete absence of invasive disease in the breast (ypT0-is) and absence of involved axillary lymph nodes (ypT0-is, pN0). The latter was defined as total pCR.

### Statistical analyses

Multivariable Cox regression models [27] stratified by centre, were used to estimate adjusted hazard ratios. Where surgical covariates were included in survival analyses, landmark analysis (time from surgery) was used. Multivariable logistic regression was used to estimate adjusted odds ratios for the relationship between baseline covariates and pCR. For all multivariable analysis a stepwise model selection procedure using Akaike's Information Criterion [28] was used to identify covariates to be included alongside treatment in the final model. The candidate variables for analysis from diagnosis and pCR were the baseline covariates; age, tumour size (mm), tumour grade and ER status. Landmark analysis [29] included further surgical covariates; tumour size (post-op), chemotherapy regimen, pCR status and surgery type. Once a final model was identified, various two-way interaction terms were explored and retained if *p* < 0.05.

### Statistical analyses

Categorical variables are presented as frequency (percentage) and continuous variables as mean (SD). Survivor functions were estimated using the Kaplan-Meier method [30] and compared using the log-rank test [31], stratifying by centre. Multivariable Cox regression models stratified by centre, were used to estimate adjusted hazard ratios. Where surgical covariates were included in survival analyses, landmark analysis (time from surgery) was used. Multivariable logistic regression was used to estimate adjusted odds ratios for the relationship between baseline covariates and pCR. For all multivariable analysis a stepwise model selection procedure using Akaike's Information Criterion was used to identify covariates to be included alongside treatment in the final model. The candidate variables for analysis from diagnosis and pCR were the baseline covariates; age, tumour size (mm), tumour grade and ER status. Landmark analysis (3) included further surgical covariates; tumour size (post-op), chemotherapy regimen, pCR status and surgery type. Once a final model was identified, various two-way interaction terms were explored and retained if *p* < 0.05. All statistical analyses were conducted using Stata 13 (StataCorp. 2013).
